# Dietary *Bacillus* spp*.* supplementation to both sow and progenies improved post-weaning growth rate, gut function, and reduce the pro-inflammatory cytokine production in weaners challenged with *Escherichia coli K88*

**DOI:** 10.1186/s42523-024-00290-y

**Published:** 2024-01-24

**Authors:** Vetriselvi Sampath, Sungbo Cho, Jinuk Jeong, Seyoung Mun, Choon Han Lee, Rafael Gustavo Hermes, Apichaya Taechavasonyoo, Natasja Smeets, Susanne Kirwan, Kyudong Han, In Ho Kim

**Affiliations:** 1 Department of Animal Resource and Science, Dankook University, No. 29 Anseodong, Cheonan, Chungnam 330-714 South Korea; 2 Department of Bioconvergence Engineering, Dankook University, Jukjeon, 16890 Republic of Korea; 3 Department of Microbiology, College of Science and Technology, Dankook University, Cheonan, 31116 Republic of Korea; 4 Center for Bio-Medical Engineering Core Facility, Dankook University, Cheonan, 31116 Republic of Korea; 5 Kemin Industries Inc Headquarters, 1900 Scott Ave Des Moines, Des Moines, IA 50317 USA

**Keywords:** Probiotics, Growth performance, Gut microbiome, Cytokine immune response, Weanling pig

## Abstract

**Background:**

The use of probiotics (PRO) in late gestation sow and their impact on progenies’ performance during the post-weaning stage has received more attention from the researchers recently. This study aimed to analyze the effect of probiotic mixture (*Bacillus subtilis* and *Bacillus licheniformis*) on both sow and offspring’s performance.

**Methods:**

First experiment (Exp.1) was conducted from the 100th day of gestation through to post-weaning. A total of twenty sows and their litters were assigned to one of two dietary treatments, Control (CON) based diet and PRO− CON+ 0.05% probiotic mixture. Dietary treatments were arranged in a split-plot pattern with sow and weaner treatment (CON and PRO diet) as the main and sub plot. Exp.2. *E. coli* challenge study was carried out two weeks after weaning with 40 piglets. Dietary treatments remained same while all pigs were orally administered with a 1.5 ml suspension of 1010 CFU of K88 strain of *E. coli* per ml.

**Result:**

PRO group sow showed significantly decreased backfat thickness difference and body weight difference after farrowing and at the end of weaning d21. The nutrient digestibility of PRO group sows was significantly higher at the end of weaning. Moreover, piglets born from PRO group sow showed higher weaning weight and tend to increase average daily gain at the end of d21. The addition of mixed probiotic in sow and weaner diet had suppressed the production of TNF-α and interleukin-6 in *E. coli* challenged pigs. The phyla *Firmicutes* and *Bacteroidetes* in *E. coli -*challenged pigs were highly abundant while, the relative abundance of *clostridium_sensu_stricto_1* at genus level was significantly reduced by the inclusion of probiotic in both the sow and weaner diet. Also, taxonomic distribution analysis showed significantly lower prevalence of *Clostridium* and *Brachyspira* and higher prevalence of *Lactobacilli* in *E. coli-*challenged pigs that were born from PRO group sow and fed CON and PRO weaner diet.

**Conclusion:**

This study reveals that the inclusion of 0.05% mixed probiotics (*Bacillus* spp.) to both sow and their progenies diet would be more beneficial to enhance the post-weaning growth rate, gut health, and immune status of *E. coli* challenged pigs.

**Supplementary Information:**

The online version contains supplementary material available at 10.1186/s42523-024-00290-y.

## Introduction

Gestation, parturition, and lactation periods are the main stages in pig production [1]. During these stages’ sows may undergo physical and metabolic changes to meet their nutritional and energy requirements [2]. Indeed, nutritional imbalance during gestation may affect the reproduction performance of sows and also affect the weaning weight of their progeny’s [3]. Thus, maintaining a healthy metabolism and reducing inflammation in sows during late gestation period has become important strategy to ensure their performance and progeny growth. To date, several approaches have been implemented to improve the health of sows and their young one. For instance, antibiotic growth promoters (AGP) and pharmacological levels of Zinc Oxide (ZnO) have been used to reduce the inflammation [4] and reduce the diarrhea incidence in piglets however, the spread of antibiotic residues, and interruption of gut microbiota homeostasis has become an alarming concern recently [5]. Thus, many countries including EU and Republic of Korea has prohibited to use certain AGP (since, 2003) and ZnO (since, 2022) in pigs’ diet [6, 7]. This sudden restriction has driven the researchers to find an array of specialty feed ingredients and/or in-feed additives. Concurrently, prebiotics, probiotics, and organic acids took the top spot for supporting the intestinal epithelial integrity and immune-modulating agents in livestock.

Probiotics, a live microorganism can be formulated in many types including foods, drugs, and dietary supplements [8]. Such probiotics have been used in sows’ diet for their beneficial effects on their well-being health promotion and reproductive performance [9]. The innate immunity of pregnant sows is often impaired, but the administration of probiotics to sows revealed better health status, colostrum quality, and piglet performance [10, 11]. Previously, Liu et al. [12] reported that sows fed complex probiotics during late gestation has improved “colostrum quality, shorten estrus intervals, reduce serum inflammatory factors, and increase piglet weaning weight by reducing the diarrhea rate. Similarly, Xiang et al. [13] demonstrate that inclusion of *Clostridium butyricum* and *Saccharomyces boulardii* to gestation sows had modulate the gut microbiota diversity and community structure of both sow and piglets by reducing the abundance of pathogenic bacteria, such as *Salmonella, Clostridium, and Escherichia coli*. Generally, *Bacillus* spp*.* have a strong scientific data that substantiates the validity of their use as preferred probiotics [14]. Recent studies demonstrate that *Bacillus subtilis* has various mechanisms of action including antimicrobial effect by synthesis of antimicrobial substances, antidiarrheal effect, immunostimulatory effect, competitive exclusion of pathogens, prevention of intestinal inflammation, and normalization of intestinal microbiota [15]. In 2015, Cai et al. [16] reported that dietary supplements with *Bacillus* spp. improved growth performance, villi length of the duodenum and jejunum,and reduce the diarrhea rate in nursery pigs.

The intimate contact between sows and their offspring’s is more important to determine the early bacterial colonization of the gastrointestinal tract [17] that exerts “microbial imprinting” (long-term effect) [18]. Besides, postweaning diarrhea (PWD) which occurs during the first two weeks after weaning [19] has become an emerging issue worldwide over the decades as it creates welfare issues and poor performance that resulted with negative economic impression [20]. *Escherichia coli* (*E. coli*) is a common cause of PWD, particularly fimbriae F18 and F4 (K88) are considered to be common pathogenic strains [19]. Transmission of good and bad bacteria from mother to offspring occurs in the reproductive tract during farrowing time when young piglets comes into contact with milk and lick the feces during lactation [21]. But the balance between these good and pathogenic bacteria can be altered from post farrowing to weaning [22]. Earlier studies pointed that dietary strategies can regulate the bacterial populations in sows and confer direct health benefits to their offspring [23]. For example, Baker et al. [23] and Starke et al. [24] reported that sows fed diet supplement with probiotic has a positive influence on the intestinal microbiota of suckling pigs and that effect seems to extended during post-weaning stage [25]. Similarly, Pan et al. [26] demonstrate that dietary supplement with *S. cerevisiae* and *Bacillus licheniformis* had significantly increased the relative abundance of *Lactobacillus* and reduced *E. coli* in LPS challenged piglets. Based on these literatures, we intended to do comprehensive research on the prolonged effect of adding *Bacillus* spp*.* supplement to sow and offspring. Cytokines plays an important role in regulating the immune and inflammatory responses and the gut barrier function [27]. Whilst, pro-inflammatory cytokines IL-6 and TNF-α play a dominant role in the cell-mediated immune response, and participate in the maintenance of tissue integrity [28]. Such, TNF-α, can increase intestinal permeability through the dysregulation of tight junction proteins [29]. The inflammatory and acute phase responses after challenge with endotoxin materials has been shown in several studies [30, 31] yet to the best of our knowledge the potential effect of *Bacillus subtilis and Bacillus licheniformis* on the production of cytokines particularly, TNF-α, and Interleukin-6 in *E. coli K88* challenged weaning pigs is still not well elucidated.

From the above-mentioned literature [23, 24] we hypothesize that the addition of probiotics to sow and progenies with same spp. might be beneficial to enhance their performance. So far, single strain probiotics are commonly used to enhance animal performance, but recent days the vast majority of novel probiotics are used as multi-strain. Herein, we focus to use triple-strain **(***Bacillus* spp.—ATCC PTA-6737, PTA-127113 and PTA-127114*)* probiotic which was commercially prepared with the name of ENTEROSURE™ and obtained from © Kemin Industries, Inc. (USA). Though the administration of probiotics in swine has been investigated widely, the conclusive data on use of triple strain *Bacillus* spp. to both sow and their progenies with same dosage from late gestation to post-weaning does not exist. Thus, we intend to evaluate the potential effect of feeding *Bacillus* spp*.* on the reproduction performance of sow and their progenies growth performance and nutrient digestibility (Exp 1), the gut barrier function, and pro-inflammatory cytokine immune response of pigs challenged with *E. coli* K88 strain (Exp 2).

## Results

### Exp. 1

#### Reproductive performance of sows

Sow-fed diet supplement with mixed probiotics has significantly decreased backfat thickness loss (BFTL) (*P* = 0.056) during post farrowing and body weight loss (BWL) (*P* = 0.017) at the end of weaning d 21. However, both CON and probiotic group sows showed no improvements in their litter size, body condition score, and average daily feed intake (ADFI) Also, sows fed 0.05% probiotics showed significantly increased nutrient digestibility dry matter (DM) (*P* = 0.0003), nitrogen (N) (*P* =  < 0.0001), and gross energy (GE) (*P* =  < 0.0001) compared to those fed CON diet at the end of weaning d 21 (Table 1). Though there were no differences (*P* > 0.10) found in litter size, piglets born from PRO group sows showed higher weaning weight (*P* = 0.026) and tend to increase average daily live weight gain (*P* = 0.096) during the 21-days of suckling period (Table 2).

**Table 1:** Supplemental effect of mixed probiotics on reproduction performance and nutrient digestibility in sow^1^

Items	CON	PRO	SEM^2^	*P* value
Number of sows	10	10	**–**	**–**
Parity	3.4	3.4	0.3	0.271
Litter size				
Total born, head	11.5	11.9	0.5	0.367
Total alive, head	11.1	11.6	0.5	0.242
Stillbirth, head	0.3	0.1	0.1	0.075
Mummification, head	0.1	0.2	0.1	0.720
Survival rate^3^, %	94.44	97.80	1.68	0.635
Sow body weight, kg				
Initial	209.1	222.1	8.0	0.591
Pre-farrowing	233.3	244.7	7.2	0.538
Post-farrowing	213.1	226.1	7.8	0.608
Weaning	194.6	208.8	7.5	0.545
BW change 1^4^	24.2	22.6	1.4	0.901
BW change 2^4^	20.1	18.7	1.0	0.824
BW change 3^4^	18.5^a^	17.3^b^	0.5	0.017
Backfat thickness, mm				
Initial	19.5	18.9	0.4	0.466
Pre-farrowing	21.0	20.0	0.3	0.195
Post-farrowing	19.5	18.8	0.3	0.409
Weaning	17.4	16.8	0.4	0.714
BFT change 1^5^	1.5	1.1	0.2	0.622
BFT change 2^5^	1.5^a^	1.2^b^	0.1	0.056
BFT change 3^5^	2.1	2.0	0.3	0.563
Body condition score				
Initial	2.9	3.0	0.1	0.306
Pre-farrowing	3.3	3.3	0.1	0.824
Post-farrowing	3.2	3.1	0.1	0.978
Weaning	2.8	2.8	0.2	0.987
Average daily feed intake, kg				
Pregnant	2.58	2.59	0.02	0.476
Lactation	7.23	7.26	0.01	0.560
Estrus interval, d	4.5	4.3	0.4	0.939
Weaning, d 21				
Dry matter	61.65^b^	63.45^a^	1.89	0.0003
Nitrogen	60.30^b^	61.49^a^	0.32	< 0.0001
Energy	61.49^b^	62.85^a^	0.30	< 0.0001

^1^CON, Basal diet; TRT, Basal diet + 0.05% mixed probiotics

^2^Standard error of means

^3^Survival rate (at birth and weaning)

^4,5^BW and BFT change: (1) Initial to pre-farrowing; (2) pre-farrowing to post-farrowing (3) post-farrowing to weaning

^a,b^Means in the same row with different superscripts differ significantly (*P* < 0.05)

**Table 2:** Supplemental effect of mixed probiotics on suckling piglets’ performance^1^

Items	CON	PRO	SEM^2^	*P* value
Suckling’s/litter				
INO- d 0 (start, foster)	11.1^b^	11.6^a^	0.10	0.104
FNO- d 21 (weaning)	10.7	11.3	0.20	0.408
Survival % from d 0–21	96.36	97.42	1.76	0.634
Body weight, kg				
Birth weight	1.51	1.55	0.02	0.166
Weaning	6.07^b^	6.42^a^	0.07	0.026
Average daily gain, g				
Overall (d1–21)	216^b^	231^a^	3	0.096

^1^CON, Basal diet; TRT, Basal diet + 0.05% mixed probiotics. INO-Initial number of suckling (born alive); FNO-final number of suckling (weaned/sow)

^2^Standard error of means

^a,b^Means in the same row with different superscripts differ significantly (*P* < 0.05) and trends (*P* < 0.10)

#### Weaner performance

The growth performance and nutrient digestibility of pigs from 0 to 6 weeks of age after weaning in relation to respective sow treatment and weanling dietary treatments was shown in Table 3. Significant increases in average daily gain were recorded during 2–6 weeks and the entire post-weaning period (i.e., 0–6 weeks). In addition, compared with the control treatment the growth rate of was significantly increased when both sows and weanlings received probiotics. However, over the entire 6-weeks, the increases in growth rate due to probiotic inclusion in weanling diets were significant whether sows had, or had not, received probiotics. When comparing the control group (no probiotics in sow feeds or weanling feed) with probiotic inclusion in both sow and weanling, probiotic group showed an increased growth rate (37 g/animal/day) (+ 6.9%, *P* < 0.05) from 0 to 6 weeks of age and a 30 g/head/day (+ 6.5%, *P* < 0.05) increase across the post-weaning period. Moreover, the positive post-weaning growth rate responses were supported by improvements in DM and N digestibility. The inclusion of probiotics in both sow and weanling diets showed increased DM digestibility from 80.08 to 82.59% (*P* < 0.05) and nitrogen digestibility from 78.34% to 79.76% (*P* < 0.05) at the end of week 6. However, there were no effects found on BW, ADFI, and gain to feed ratio (G: F), and nutrient digestibility of N and E in weanling. In addition, the fecal score of weaning pigs was not affected (*P* > 0.10) either by main effects of sow dietary treatment or by sub effect of weaner dietary treatment (data not included).

**Table 3:** Supplemental effect of mixed probiotics on sow and weaning dietary treatment on the growth performance and nutrient digestibility of weaning pigs^1,2^

Item				
Sow treatment^3^	CON		PRO	
Weaner treatment^4^	CON	PRO	CON	PRO
Body weight, kg				
Initial	6.39 ± 0.20^b^	6.39 ± 0.20^b^	6.48 ± 0.22^a^	6.48 ± 0.21^a^
Week 2	10.74 ± 0.54^b^	10.98 ± 0.48^ab^	10.92 ± 0.48^ab^	11.09 ± 0.50^a^
Week 6	25.79 ± 0.80^c^	26.68 ± 0.79^ab^	26.22 ± 0.72^bc^	27.16 ± 0.79^a^
Weeks 0–2				
ADG, g	311 ± 24.68	327 ± 20.04	317 ± 19.24	330 ± 20.90
ADFI, g	444 ± 36.48	461 ± 27.95	455 ± 30.47	465 ± 31.04
G: F	1.426 ± 0.01	1.41 ± 0.01	1.43 ± 0.015	1.408 ± 0.012
Weeks 2–6				
ADG, g	537 ± 11.42^b^	561 ± 12.74^ab^	547 ± 12.55^b^	574 ± 12.60^a^
ADFI, g	912 ± 24.82	941 ± 26.92	922 ± 30.06	958 ± 29.24
G: F	1.695 ± 0.01	1.676 ± 0.01	1.685 ± 0.018	1.668 ± 0.014
Overall				
ADG, g	462 ± 14.63^c^	483 ± 14.33^ab^	470 ± 12.44^bc^	492 ± 13.91^a^
ADFI, g	756 ± 26.56	781 ± 25.52	766 ± 25.94	794 ± 26.57
G: F	1.635 ± 0.009	1.616 ± 0.005	1.628 ± 0.014	1.611 ± 0.011
Nutrient digestibility				
Week 6				
Dry matter	80.08 ± 0.75^b^	82.34 ± 0.49^ab^	81.34 ± 0.95^ab^	82.59 ± 0.80^a^
Nitrogen	78.34 ± 0.69^b^	79.31 ± 0.98^ab^	78.9 ± 1.25^ab^	79.76 ± 0.72^a^
Energy	81.1 ± 0.73	81.48 ± 0.94	81.36 ± 0.87	81.65 ± 0.52

^1^A total of 200 piglets which weaned from CON and PRO group sows were assigned to pens and pens were dispensed to dietary treatments in a spilt-plot pattern. ^2^Sow and weaning dietary treatments (CON and PRO diet) were served as the main and sub-plots respectively

^3,4^Sow and weanling dietary treatment consisted of corn soybean mean-based control diet and control diet supplemented with 0.05% of *Bacillus subtilis* (FXA and PB6) and *Bacillus licheniformis* probiotics

^a,b^Means in the same row with different superscripts differ significantly (*P* < 0.05)

### Exp. 2

#### Performance of weaning pigs experimentally infected with *Escherichia coli* K88

Table 4 shows the effect of probiotics supplementation on the growth performance and cytokine level in weanling pigs challenged with *E. coli* K88. The piglets born from PRO group sows and had 0.05% probiotic showed a heavier average body weight (± 1.79 kg) at the end of week 6 (i.e., 24.45 kg versus 26.24 kg, *P* < 0.05). In addition, average daily gain (ADG) (*P* < 0.05) was significantly increased (297 g versus 313 g, *P* < 0.05) in *E. coli* challenged pigs that were born from PRO group sows and had 0.05% probiotic from 2 to 6 weeks after weaning. The inclusion of probiotics in both mother and weaners diet had reduced (*P* < 0.05) the production of interleukin-6 and TNF-α in *E. coli-*challenged pigs.

**Table 4:** Supplemental effect of mixed probiotics on sow and weaning dietary treatment on the growth performance and cytokines response in weaning pigs challenged with *E. coli* K88^1,2^

Items				
Sow treatment^3^	CON		PRO	
Weaner treatment^4^	CON	PRO	CON	PRO
Body weight, kg				
Initial	6.31 ± 0.49	6.31 ± 0.48	6.34 ± 0.43	6.34 ± 0.47
Week 2	10.47 ± 0.37	10.62 ± 0.54	10.59 ± 0.45	10.72 ± 0.52
Week 6	24.45 ± 0.54^c^	25.75 ± 0.21^ab^	25.09 ± 0.68^bc^	26.24 ± 0.318^a^
Weeks 0–2				
ADG, g	297 ± 8	308 ± 4.07	304 ± 2.35	313 ± 3.57
ADFI, g	395 ± 8.49	404 ± 0.295	401 ± 15.72	410 ± 4.38
G: F	1.329 ± 0.007	1.313 ± 0.018	1.321 ± 0.06	1.31 ± 0.0009
Weeks 2–6				
ADG, g	499 ± 5.85^b^	540 ± 11.53^ab^	518 ± 8.28^ab^	554 ± 7.39^a^
ADFI, g	870 ± 28.14	930 ± 4.63	898 ± 5.18	948 ± 31.07
G: F	1.744 ± 0.07	1.722 ± 0.04	1.734 ± 0.017	1.709 ± 0.03
Overall				
ADG, g	432 ± 1.23^c^	463 ± 6.33^ab^	447 ± 4.73^bc^	474 ± 3.73^a^
ADFI, g	712 ± 21.59	754 ± 2.99	732 ± 8.69	769 ± 19.25
G: F	1.648 ± 0.05	1.631 ± 0.02	1.64 ± 0.002	1.621 ± 0.02
*Cytokine*				
TNF-α (pg/ml)				
Before K88	51.95 ± 5.91	43.15 ± 4.87	47.11 ± 3.44	45.27 ± 4.56
After K88	57.92 ± 6.11	47.65 ± 5.04	52.46 ± 3.20	48.98 ± 4.94
Difference	5.97 ± 1.14	4.5 ± 0.677	5.36 ± 0.74	3.71 ± 0.96
Interleukin-6 (pg/ml)				
Before *K88*	263.05 ± 17.28	261.48 ± 10.85	255.24 ± 13.55	266.16 ± 13.22
After *K88*	274.24 ± 17.46	268.23 ± 9.53	265.14 ± 13.43	272.05 ± 13.27
Difference	11.19 ± 1.86	6.75 ± 1.70	9.91 ± 1.47	5.89 ± 1.61

^1^A total of 40 crossbred weaning piglets weaned from CON and PRO group sows were used for *E. coli* challenge study (two weeks later)

^2^Weanlings were assigned to pens and pens were dispensed to dietary treatments in a spilt-plot pattern. Sow and weaning dietary treatments (CON and PRO diet) served as main and sub-plot respectivelyt

^3,4^Sow and weanling dietary treatment consisted of corn-soybean mean-based control diet and control diet supplemented with 0.05% of *Bacillus subtilis* (FXA and PB6) and *Bacillus licheniformis* probiotics. Additionally, weanlings were orally administered with a 1.5 ml suspension of 1,010 colony-forming units (CFU) of K88 strain of *E. coli* per ml

^a,b^Means in the same row with different superscripts differ significantly (*P* < 0.05)

#### Fecal microbiota

**Fig. 1:**
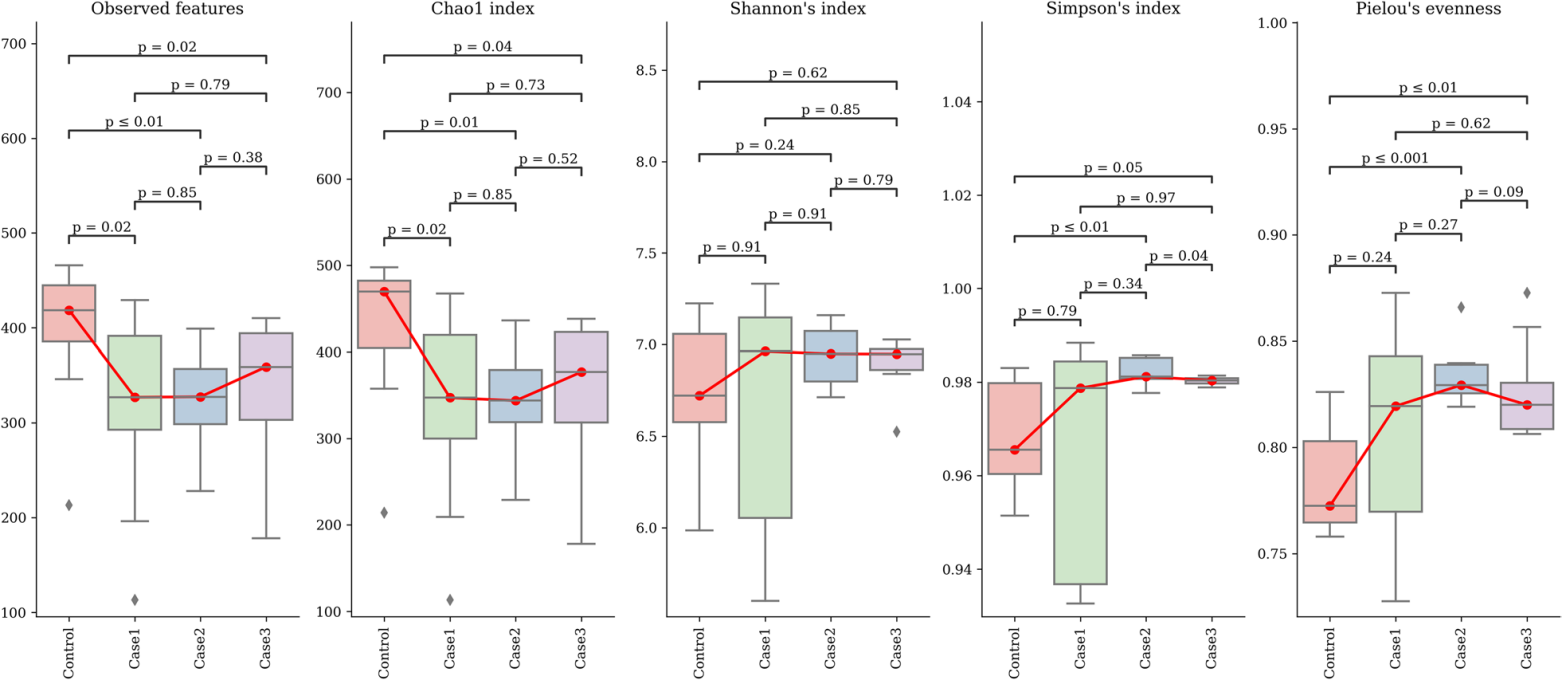
illustrate the Alpha-diversity analysis of CON (Piglets born from CON group sows and fed CON weaning diet); Case 1 (Piglets born from CON group sows and fed a diet supplemented with 0.05% probiotic); Case 2 (Piglets born from probiotic group sow and fed a weaning CON diet), Case 3 (Piglets born from probiotic group sows and a fed diet supplemented with 0.05% probiotic) pigs. Each alpha-diversity was calculated using 1: Observed_ASVs, 2: Chao 1, Shannon_index, Simpson’s index, and 5: Pielou_evenness indices in order

**Fig. 2:**
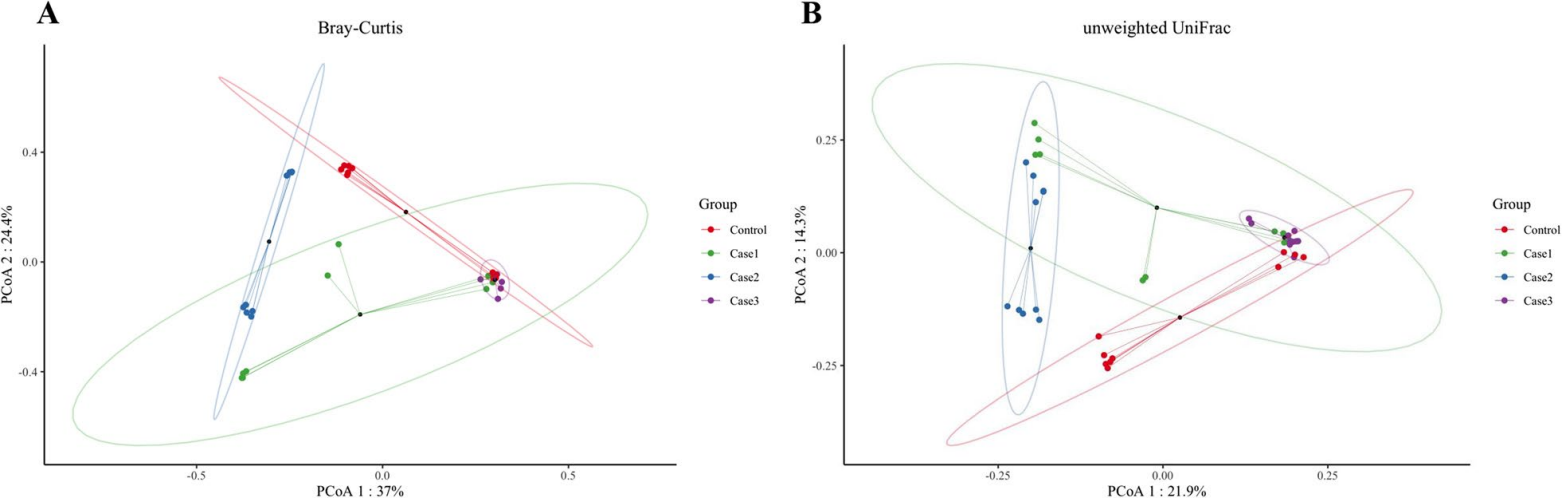
illustrate the Beta-diversity analysis of four group piglets. Microbial beta-diversity analysis measured by both Bray–Curtis distance (**a**) and unweighted UniFrac (**b**) distance matrix for all samples

**Fig. 3:**
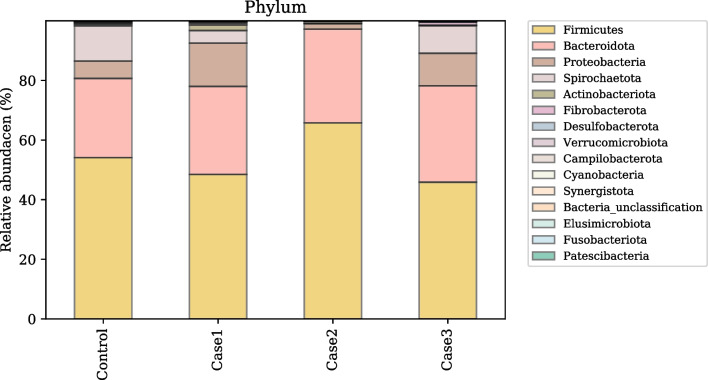
Microbial taxonomic profiles from the gut of CON (Piglets born from CON group sows and fed CON weaning diet); Case 1 (Piglets born from CON group sows and fed a diet supplemented with 0.05% probiotic); Case 2 (Piglets born from probiotic group sow and fed a weaning CON diet), Case 3 (Piglets born from probiotic group sows and a fed diet supplemented with 0.05% probiotic) pigs at the phylum levels, classified by the representation of > 1% of total sequences. Taxonomic compositions of the gut microbiota of control and other treatment groups were compared based on the relative abundance

**Fig. 4:**
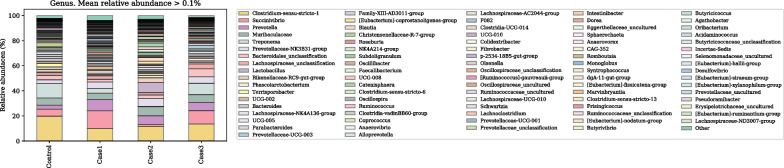
Microbial taxonomic profiles from the gut of CON (Piglets born from CON group sows and fed CON weaning diet); Case 1 (Piglets born from CON group sows and fed a diet supplemented with 0.05% probiotic); Case 2 (Piglets born from probiotic group sow and fed a weaning CON diet), Case 3 (Piglets born from probiotic group sows and a fed diet supplemented with 0.05% probiotic) pigs at the genus levels, classified by the representation of > 1% of total sequences. Taxonomic compositions of the gut microbiota of control and other treatment groups were compared based on the relative abundance

The Richness and Chao 1 indices showed significantly less diversity of microbiota in samples from the treatments that included probiotic compared with the control diets (Kruskal–Wallis *P* < 0.05), there were no significant differences observed from Shannon’s and Simpson’s indices of diversity (Kruskal–Wallis *P* > 0.05). However, Pielou’s evenness index showed significantly higher uniformity of species within the samples for the treatments that included probiotic (Kruskal–Wallis, *P* < 0.05) which indicates good balance and stability of the microbiota (Fig. 1). There were significant differences in the microbial composition between the treatment groups (*P* < 0.05) for the Bray–Curtis and UniFrac indices for the treatment comparisons (Fig. 2). *Escherichia coli-*challenged pigs that were born from PRO group sow and fed CON diet has highly abundant phyla *Firmicutes* and *Bacteroidetes*, and were represented by 54.78% and 30.50% of other sequences, respectively (Fig. 3). The next most abundant phylum was *Proteobacteria* and *Spirochaetota,* which was represented by 7.50 and 6.22% of the total sequences; the remaining phyla were each represented by < 1.00% of all sequences. Some taxa were not classified at the phylum level but showed high abundance at the genus level. The relative abundance of *clostridium_sensu_stricto_1* in the genus level were significantly reduced by the inclusion of probiotic in both sow and weaner diet. The next most abundant genus was *Succinvibrio* (14.12%), and *Treponema* (11.50%), *Prevotella* (8.8%), *Muribaculaceae* (7.33%), and *Prevotelleceae*_NK3B31_ group (6.46%) (Fig. 4)*.* Taxonomic distribution analysis (LEfSe) showed significantly lower prevalence of Clostridium and *Brachyspira* and a higher prevalence of Lactobacilli in *E. coli-*challenged pigs that were born from PRO group sow and fed CON and PRO weaner diet (Table 5).

**Table 5:** *Clostridia, Brachyspira*, and *Lactobacilli* prevalence and treatment differences in fecal microbiota

Species	Prevalence, %				*P*-values (LEfSe test)			
	TRT 1	TRT 2	TRT 3	TRT 4	All TRT	TRT 1v2	TRT 1v3	TRT 1v4
*Clostridia*	19.77	10.04	11.58	13.56	0.024	0.011	0.026	0.054
*Brachyspira*	11.51	3.81	0.15	8.92	0.000	0.000	0.000	0.021
*Lactobacilli*	0.54	0.85	8.09	1.40	0.000	0.733	0.000	0.005

TRT 1: Piglets born from CON group sows and fed CON weaning diet

TRT 2: Piglets born from CON group sows and fed a diet supplemented with 0.05% probiotic

TRT 3: Piglets born from probiotic group sow and fed a weaning CON diet

TRT 4: Piglets born from probiotic group sows and a fed diet supplemented with 0.05% probiotic

Addition to diets all weanlings were orally administered with a 1.5 ml suspension of 1010 CFU of K88 strain of *E. coli* per ml

## Discussion

### Exp. 1

Feed consumption during lactation is critical to achieve milk production and to support large litters with minimal utilization of sow body reserves [32]. Previously, Bohmer et al. [33] reported that the inclusion of *Bacillus* spp*.* to sows’ diet during late gestation and lactation had significantly increase their feed intake, and this statement was not agreed with the current study in which sows fed diet supplemented with neither CON nor 0.05% probiotic supplement showed no improvement in their feed intake. Generally, when nutrient digestibility reduce, sows may consume larger amounts of feed to meet their nutrient requirements, resulting in increased feed intake. Hence, we supposed that no improvements in daily feed intake of sow might be due to increased nutrient utilization. Contrast to the current finding, Kritas et al. [34] noted a significant improvement in sow body condition score during gestation and at the time of farrowing with probiotic supplement. In 2016, Hayakawa et al. [35] reported that sows fed diet supplemented with mixed probiotics containing a *Bacillius mesentericus strain* (2.0 × 10^8^ CFU/kg of feed) improved their reproductive performance and growth performance of piglets). While, Rychen et al. [36] found no improvement in the productive performance of sow by the inclusion of *B. subtilis* PB6 (1.0 × 10^8^ CFU/kg of feed). The possible reason for this discrepancies result was likely due to the intense feeding or improper supply of nutrients during the gestation period. Previously, Tantasuparuk et al. [37] stated that excessive BWL in sow might increase the weaning to estrus intervals however, in this study the BWL of both groups remains same accordingly, weaning-to estrus interval remains similar and we suggested that probiotics supplementation might have beneficial effects on the sow reproductive performance. Nutrient digestibility plays a crucial role in the efficient utilization of feed resources in pig production. When nutrients are well digested and absorbed, they can be efficiently utilized by the pig's for various physiological functions, including muscle growth, bone development, immune function, and reproduction. Predominantly, energy utilization plays a vital role in reproductive functions, including estrus expression and ovulation. If the sow's diet is not competently digested then energy cannot be absorbed for metabolic processes thus resulting negative impact on sow reproduction performance, including decreased litter size, and extended weaning-to-estrus intervals. Fortunately, sows fed 0.05% *Bacillus* spp. showed increased nutrient digestibility of DM, N, and E compared to those fed CON diet thus resulted in better reproduction performance.

Farrowing crate provides the first place to influence the development of a piglet's microbiome, as they were entirely dependent on their mother [38]. The prenatal intestine of piglet has the ability to absorb and exploit large molecules during the final weeks of gestation, and appears about 2wk before delivery and stops within 1 to 2 d after birth [39]. Mehrazar et al. [40] stated that maternal diet has affect the intestinal closure in foetuses via the placenta and helps neonates to absorption of maternal IgG. In the earlier study, Rychen et al. [36] reported that piglets born to sows fed 1.0 × 10^8^ CFU/kg B. subtilis PB6 supplement had less weaning weight. While, Baker et al. [23] found greater weaning weight in piglets born to sows fed *Bacillius-*based direct-fed (3.75 × 10^8^ CFU/kg of feed) diets from late gestation to farrowing, and this result was constant with the present findings in which piglets born from PRO group sows showed higher weaning weight and average daily live weight gain during the 21-days of suckling period. Herein, we proposed two mechanisms for achieving higher weaning weight, the first one might be better utilization of nutrients from colostrum, which serves as important source of IgG to ensure the passive protection for suckling’s while, another mode of action could be the reduction of pathogenic bacterial presence in their intestines.

Weaning stress often lead to excessive intestinal micro-ecology, high diarrhea incidence, and subsequently lower growth rate [41]. However, these hitches can be lessened by dietary probiotics supplementation, as it can modulate the intestinal microbial population, stimulate the immune system of the host, and enhance their gut health and growth performance by reducing the diarrheal incidences [42]. Previously, Chen et al. [43] reported that pigs fed 0.2% bacillus-based probiotic had improved ADG (11%, *P* < 0.05). Similarly, Nguyen et al. [44] reported that the inclusion of 0.3% of mixed probiotic (1 × 10^12^ CFU kg^−1^ of *B. coagulans,* 5 × 10^11^ CFU kg^−1^ of *B. licheniformis,* 1 × 10^12^ CFU kg^−1^ of *B. subtilis* and 1 × 10^11^ CFU kg^−1^ of *C. butyricum*) linearly increased the growth performance of weaning pigs and this result were constant with the present findings. Although daily gain showed improvements in pigs fed a 0.05% probiotics supplement, the BW, daily feed intake and gain/feed were unaffected over 6 weeks was constant with Mun et al. [45] who found same result in weaning pigs fed diet supplemented with 0.01% *Bacillus subtilis* and *Bacillus licheniformis* probiotics. While, Sampath et al. [46] study reveals that dietary *Bacillus licheniformis* and *Bacillus subtilis* has significantly improved the growth performance of weaning pigs. The discrepancies result from the above-mentioned studies made us to hypothesized that age of pigs, species, and strain is also a considerable fact to relate the efficacy of probiotics as they are very active in animals during microbiome development and/or when microbiome stability is compromised. We supposed that reduced pathogen load in the gut may lead to improve the nutrient absorption, thus contribute to increase ADG in pigs. Generally, early weaning pigs, require a well-balanced and highly digestible diet to support their rapid growth and development. Besides, high nutrient utilization ensures that pigs can efficiently absorb the nutrients from their feed, leading to optimal feed efficiency and overall performance. Considering this fact, we infer that no improvements in the G: F could be due to less improvements in DM and N digestibility. Previously, Chen et al. [43] reported that finishing pigs fed diet contain 0.2% *Bacillus* supplement showed slightly increment in N digestibility which partially agreed with the current finding. However, Nguyen et al. [44] found no difference in nutrient retention of weaning pigs by the inclusion of mixed probiotic. Previously, Jonsson and Conway [47] addressed that *Bacillus* spp. is not normal components of indigenous intestinal microflora and they are hard to colonize in the digestive tract accordingly, we believe that this inconsistent results on nutrients digestibility could be due to same reason as cited above.

### Exp. 2

Weaning is usually associated with substantial dynamic changes of the intestinal microbiota that may affect intestinal functions [48]. While, probiotics treatment may induce beneficial effects by acting on the intestinal ecosystem [49]. Among many bacterial species *Bacillus subtilis* acts as a facultative anaerobic agent and widely used as a potential candidate in monogastric animal diet. Colonization of the inoculated probiotic strains is not always considered crucial, since probiotics can impact the host through beneficial modulation of the gut microbial community and/or by enhancing their immunological system [50]. In this regard, early application of probiotics to weaned pigs is believed to promote their resilience to overcome post-weaning difficulties. Our findings showed that inclusion of probiotics in both sow and offspring at early-stage aid increase ADG in piglets when they are subjected to an *E. coli* challenge. Previously, Scharek et al. [51] reported that adding *Bacillus cereus var. toyoi* probiotics in the diet of sow and piglets reveal a better intestinal immune system at d 28 of weaning and nursery phase which was agreed with present study in which Richness and Chao 1 indices showed significantly less diversity of microbiota in samples from the treatments that included probiotic compared with the control diets but there were no significant differences for the Shannon’s and Simpson’s indices of diversity [52]. While, Pielou’s evenness index showed significantly higher uniformity of species within the samples for the treatments that included probiotic which indicates good balance and stability of the microbiota. There were significant differences in the microbial composition between the treatment groups for the Bray–Curtis and UniFrac indices for the treatment comparisons. Phylum *Firmicutes* and *Bacteroidetes* are dominant bacteria in the porcine intestine [53] such phyla *Firmicutes* and *Bacteroidota* of were highly dominant in piglets born from PRO group sows and fed CON diet. The current finding was related with Sampath et al. [54] who found similar result in neonatal piglets fed diet supplement with *Lactobacillus sp.* Taxonomic distribution analysis (LEfSe) showed significantly lower prevalence of *Clostridia* and *Brachyspira* and higher prevalence of *Lactobacilli* in *E. coli-*challenged pigs that were born from PRO group sow and fed CON and PRO weaner diet. Herein, the increased ADG finding could be due to the modification of the microbiota ecosystem (higher *Lactobacilli* prevalence and decreased *Clostridia* and *Brachyspira*) and/or due to the effect of *B. subtilis* spp. which have antimicrobial effects against broad spectrum of pathogens.

Cytokines are immunoregulatory peptides that contribute to innate and adapt immunity by playing an essential role in immunoregulation [55]. Previously, Cao et al. [56] reported that *Bacillus amyloliquefaciens* SC06 (BaSC06) supplement alleviate the intestinal inflammation of fattening pigs by regulating the expression of IL-6, IL-8 and MCP1 pro-inflammatory cytokines in the intestinal mucosa. Following this, Wang et al. [57] stated that weaned piglets fed diet supplemented with *Lactobacillus* fermentum and *Pedioccocus acidilactici* reduced the concentration of IL-6, IL-1β, IFN-γ in the serum thereby reducing the damage caused by inflammation and this finding correlates with the present study in which weanlings receive probiotic diet highly suppress the production of serum interleukin-6 and TNF-α concentration in *E. coli* challenged pigs. It should be noted that *E. coli* has molecular patterns on its surface, such as lipopolysaccharides (LPS) and flagellin that bind with toll-like receptors on macrophage surfaces, and triggers to secrete interleukins (IL-1, IL-6, and IL-8) [58]. Therefore, it can be suggested that feeding probiotic to maternal sow might help their offspring to modulate the secretion of cytokines in response to endotoxin challenge such as *E. coli* by increase the chance of establishing a homeostatic ecosystem of the host to withstand diarrhea at postweaning stage.

## Conclusion

Our study demonstrates that feeding mixed probiotics diet to sows could supplement significantly decreased the backfat thickness loss and body weight loss after farrowing and at the end of weaning d 21, respectively. Also, increased the nutrient retention. Moreover, piglets born from PRO group sows exhibit higher weaning weight and average daily live weight gain at end of suckling (d21) as well as post-weaning. Furthermore, PRO group piglets showed increased dry matter and nitrogen retention at the end of week 6. The inclusion of probiotics in both mother and weaners diet significantly suppress the production of interleukin-6 and TNF-α in *E. coli-*challenged pigs and protect them from post weaning diarrhea. The phyla *Firmicutes* and *Bacteroidetes* were highly abundant in *E. coli-*challenged pigs that were born from PRO group sow and had CON diet. Whereas, the relative abundance of *clostridium_sensu_stricto_1* in the genus level were significantly down- regulated by the inclusion of probiotic in both sow and weaner diet. Also, taxonomic distribution analysis (LEfSe) showed significantly lower prevalence of *Clostridia* and *Brachyspira* and higher prevalence of *Lactobacilli* in *E. coli-*challenged pigs that were born from PRO group sow and fed CON and PRO weaner diet. Collectively, these findings suggest that adding 0.05% *Bacillus* spp. to sow from the 100th day of gestation through to weaning, and subsequent feeding to their sucklings’ with same *spp*. would be beneficial to enhance both sow-progenies performance and to improve the gut function and immune status of weaners challenged with *E. coli* K88. Also, we believe that this novel study would provide a new perception for the cost-effective pig production.

## Methods

### Animals, study design, feed, and feeding schedule

#### Exp. 1-sow and their offspring’s

A total of twenty sows (Duroc mated Landrace x Yorkshire) with an average parity of 3.4 (ranging from 1 to 4) were moved to hygienic farrowing crates (55 × 80 inch) 2 weeks prior to farrowing and stay there until weaning d 21. These sows were split into two treatment groups i.e., CON (control) and PRO (probiotic). Daily feed allowance of sows from the 100th day of gestation to farrowing was 2.7 kg/d, whereas lactating sows had ad libitum feed intake from farrowing to weaning d21. From the 100th day of gestation through to weaning dietary treatments were incorporated, ten sows in the CON group were fed only a corn-soybean meal-based basal diet, while ten sows in the PRO group received basal diet supplemented with 0.05% of mixed probiotics (*Bacillus* spp*.*). The formulation and nutrient specifications of the gestation and lactation sow diet (mash form) are shown in Table 6 [59]. Progenies were cross-fostered within sow treatment group to equalize litter size within 24 h of birth and had free access to water and milk no creep feeding was provided.

**Table 6:** Ingredient composition of gestation diets as-fed basis

Items	Gestation	Lactation
Ingredient, %		
Corn	42.52	43.20
Wheat	22.00	23.00
Wheat bran	10.00	8.31
Soybean hull	6.20	–
Rice bran	–	2.00
Palm kernel meal	2.00	–
Soybean meal	3.00	4.10
Dehulled soybean meal	5.94	12.50
Soybean oil	1.52	2.00
Tallow	–	1.08
Molasses	3.00	–
Limestone	1.18	1.46
MCP	0.74	0.59
Salt	0.50	0.50
Methionine (98%)	0.02	0.50
Threonine (98%)	0.09	0.05
Lysine (25%)	0.59	0.18
Choline (50%)	0.15	0.12
Vitamin/mineral mixture^a^	0.55	0.40
Phytase	–	0.01
Total	100.00	100.00
Calculated value		
DE (kcal/kg)	3260	–
ME (kcal/kg)	3020	3300
C. Protein (%)	13.00	16.50
C. Fat (%)	3.80	5.69
C. Ash (%)	4.90	–
C. Fiber (%)	5.80	–
FAT %	–	5.69
Lysine (%)	0.68	0.86
Calcium (%)	0.75	0.75
Phosphorus (%)	0.50	0.56

^a^Provided per kg of complete diet: 16,800 IU vitamin A; 2,400 IU vitamin D3; 108 mg vitamin E; 7.2 mg vitamin K; 18 mg Riboflavin; 80.4 mg Niacin; 2.64 mg Thiamine; 45.6 mg D-Pantothenic; 0.06 mg. Cobalamine; 12 mg Cu (as CuSO4); 60 mg Zn (as ZnSO4); 24 mg Mn (as MnSO4); 0.6 mg I (as Ca (IO3)2); 0.36 mg Se (as Na2SeO3)

### Weaning pigs

A total of 200 crossbred [(Landrace × Yorkshire) × Duroc] piglets (average initial BW 6.43 kg ± 0.58 kg) which weaned from CON and PRO group sow were separated (n = 160) and housed in an environmentally controlled slatted plastic floor. The mechanical ventilation of the barn (1.5- × 1.5-m) was fixed at 28–30 °C. For a period of 6 weeks, all weanlings were assigned to pens and the pens were dispensed to dietary treatments in a spilt-plot pattern (Additional file 1: Fig. S1).

1. Sow dietary treatment (CON and PRO diet) served as main plot
2. Weanling dietary treatment (CON and 0.05% PRO diet) served as sub-plot

There were eight replicate/treatment with 5 (mixed sex) pigs/pen and all pigs were provided with a similar sow supplement until week 6. Sow diets and the weanling diet did not contain antibiotics or other additives with antimicrobial activity.

#### Exp. 2

At day 21, n = 40 piglets (10 pigs/ trt, without changing the group) were separated, and orally injected with 1.5 ml suspension of 1010 colony-forming units (CFU) of K88 strain of *E. coli* per ml of suspension (2 weeks) and monitored for growth rate, cytokine response, and fecal microbiota until week 6. The *E. coli* strain used in this study was prepared following the methods of Park et al. [31].

The total feeding period was divided into two stages: Weeks 0–2 as Phase I and Weeks 2–6 as Phase II. Basal diet was formulated according to the NRC recommendation [59] (Table 7) in a mash form. The nursery room was equipped with a self-feeder and stainless-steel waterer which allowed pigs to have unlimited access to feed and water.

**Table 7:** Basal diet formulations and nutrient specifications of weaning pigs (as fed basis)

Item	Weeks 0–2	Weeks 2–6
Ingredients (%)		
Corn	49.74	53.25
Soybean meal	21.78	17.82
Fermented soybean meal	4.00	3.00
Blood plasma protein	–	–
Dried Distiller’s Grain Solubles	–	10.00
Tallow	3.02	3.10
Lactose	7.78	3.18
Sugar	3.00	3.00
Whey protein	7.00	3.00
Mon-calcium phosphate	1.17	1.04
Limestone	1.12	1.28
Salt	0.10	0.10
Methionine	0.15	0.08
Lysine	0.71	0.72
Mineral mix^a^	0.20	0.20
Vitamin mix^b^	0.20	0.20
Choline (25%)	0.03	0.03
Total	100	100
Calculated value		
Crude Protein, %	18.00	18.00
Calcium, %	0.80	0.80
Phosphorus, %	0.60	0.60
Lysine, %	1.50	1.40
Methionine, %	0.40	0.35
Metabolizable Energy, kcal/kg	3400	3350
Crude fat, %	5.11	6.08
Lactose, %	12.00	5.00

^a^Provided per kg diet were: Fe, 100 mg as ferrous sulfate; Cu, 17 mg as copper sulfate; Mn, 17 mg as manganese oxide; Zn, 100 mg as zinc oxide; I, 0.5 mg as potassium iodide; Se, 0.3 mg as sodium selenite

^b^Provided per kg of diet were: vitamin A, 10,800 IU; vitamin D3, 4,000 IU; vitamin E, 40 IU; vitamin K3, 4 mg; vitamin B1, 6 mg; vitamin B2, 12 mg; vitamin B6, 6 mg; vitamin B12, 0.05 mg; biotin, 0.2 mg; folic acid, 2 mg; niacin, 50 mg; D-calcium pantothenate, 25 mg

### Sampling and clinical analysis

#### Reproductive analysis on sows

Farrowing performance was assessed by calculating the total number born, total alive, stillbirth, and mummified piglets. Also, the starter and fostered piglets until weaning were recorded to calculate the survival rate (SUR%). Individual sow’s body weight (BW, kg) was measured from 100th day of gestation (initial), pre- and post-farrowing, and at weaning to determine the body weight loss. Also, back-fat thickness (BFT, mm) (6–8 cm from the midline of the 10th rib) of each sow was measured using piglog105, SFK Tech real-time ultrasonic instrument (Herlec, Denmark) on the 107th d of gestation, pre- and post-farrowing and at weaning to determine the back-fat thickness loss. In addition, body condition score was measured at initial, pre- and post-farrowing, and at weaning (d 21). During the last 2 weeks of gestation and lactation, the feed offered, and the leftover in the feeders were calculated on a daily basis to determine the ADFI of sows. Twenty-one days after weaning, sows were taken to the breeding room (d 22) and rested for about 2 weeks. Later they were examined for standing responses caused by a back-pressure test in the presence of mature Duroc boars (twice a day) for estrus detection.

#### Nutrient digestibility

Seven days prior to fecal sampling, 0.5% chromium (III) oxide marker was added to sow diets to measure the nutrient digestibility of DM, N, and GE. Right after mixing the chromium oxide, the represented feed samples from each treatment group were collected and stored in sterilized plastic bags for further analysis. Before farrowing and at the end of weaning, approximately 100 g of fresh fecal specimens were collected from 5 sows/ treatment by rectal palpation and homogenized. Within 45 min, collected specimens were taken to the test center and stored at − 20 °C to prevent changes in nutrients. Before starting the analysis, fecal samples were kept in a WOF-L800 hot air convection drying oven (Daehan Scientific Co. Ltd, Wonju, South Korea) for 36 h at 105 °C. Next the samples were taken out from the dryer and grounded well (Willey mill, USA) to pass through a 1-mm screen sieve. Simultaneously, feed samples were also grounded. The nutrient digestibility of DM (Method 935.29), and N (method 990.03) was analyzed according to the guidelines of AOAC [60]. Chromium was determined using UV spectrophotometry (Shimadzu, UV-1201, Kyoto, Japan). Nutrient digestibility was analyzed using the following equation.

$${\text{ND(\% )}} = {1}00 - \left[ {\left( {{\text{NF}}/{\text{ND}}} \right) \times \left( {{\text{CrD}}/{\text{CrF}}} \right)} \right] \times {1}00],$$

ND, stands for nutrient digestibility, while NF, ND, CrD, and CrF stand for nutrient concentration in the feces sample, nutrient concentration in the diet, chromium concentration in the diet, and chromium concentration in the feces sample, respectively. To determine the Energy (E): 2 g (1.9980–2.0020) fecal sample was taken and placed inside the Parr 6400 (Parr instrument, Moline, IL, USA) oxygen calorimeter and run for about 7 min, the heat combustion in the samples was noted for statistical analysis. The same process was done for feed samples. Finally, nutrient digestible E was determined by: E in feed (–) E in feces.

### Suckling pig

Total pigs born alive per sow and piglets weaned per sow were recorded to determine the survival rate of offspring from birth to weaning d 21. The ADG of individual piglets (kg) was measured at birth and at weaning d 21, by calculating the difference between birth weight (kg) and weaning weight (kg)/length of the lactation period.

### Weaning pig

#### Growth performance and nutrient digestibility

The growth performance variables such as ADG, ADFI, and G: F were recorded at the end of weeks 2, 6, and the overall experimental period. Individual piglets’ BW was measured at start and end of weeks 2 and 6 using GL-6000S machine (G-Tech Inc., LTD., Seoul, South Korea) to determine their ADG. The feeder was filled in the morning (9:00 AM) and the remaining feed in the feeders were collected and weighed in the evening (5:00 PM) to calculate the ADFI. G: F was determined by dividing ADFI and ADG.

Seven days prior to fecal sampling, 0.5% chromium (III) oxide marker was added to weaning pigs’ diets. Right after mixing the chromic oxide, the representative feed samples from four treatment groups were collected and stored in sterilized plastic bags for further analysis. At the end of week 6, approximately 100 g of fresh fecal specimens were randomly collected from 2 pigs/pen (1 female and 1 male) (10:00 AM and 3:00 PM) by rectal palpation and homogenized. The nutrient digestibility of DM, N, and E was analyzed using the above-described method.

#### Fecal score

At start and end of weeks 2,3,4, and 5, the fecal score was evaluated and recorded to determine the average value of 5 pigs/pen based on Hu et al.’s [61] scoring system:1 = hard, dry pellets in a small, hard mass; 2 = hard, formed stool that remains firm and soft; 3 = soft, formed and moist stool that retains its shape; 4 = soft, unformed stool that assumes the shape of the container; 5 = watery, liquid stool that can be poured.

### Weaning pigs challenged with *E. coli* K88

#### Growth performance

The growth rate of individual pigs was recorded at start and end of weeks 2, 6, and the overall trial period to determine ADG. The ADFI and G: F was determined following the above-stated method.

#### 16S rRNA gene sequencing and microbial community analysis

At the end of week 6, approximately 300 g of fresh fecal specimens were collected from 10 piglets/treatment by rectal palpation and taken to the laboratory for further analysis. The DNA was extracted from the specimen using QiaAmp Power Fecal Kit (Qiagen, Germany).

following the manufacturer’s instructions. The purity and concentration of the genomic DNA were measured by ultra-violet spectrophotometer (Molecular Devices, USA). The V3–V4 hypervariable region of the 16S rRNA gene was performed using Illumina Miseq platform. Then the raw sequencing was processed using QIIME2 pipeline [62]. Primers and adapters were removed following the methods of Martin [63] and the sequence quality control and feature table construction were performed using Divisive Amplicon Denoising Algorithm 2 (DADA2). Phylogenetic diversity analyses were performed following the detailed methods of Quast et al. [64]. Principal coordinate analysis based on Bray–Curtis distance matrix was performed using the ‘q2-diversity’ plugin in QIIME2. Alpha diversity indices such as Chao1, Shannon, and Simpson index, and the relative abundance bar graphs were constructed using Core R Teams’ [65] ‘ggplot2’ package.

#### Immune cytokine response

0.5 ml blood was sampled from the jugular vein of each pig prior to the dosing of *E. coli K88* and after dosing (24 h). Then the collected specimens were centrifuged at 3000 g for 15 min to recover serum. Tumor Necrosis Factor (TNF)-α, and Interleukin-6 were measured using an enzyme-linked immunosorbent assay (ELISA, R&D Systems, Minneapolis, MN, USA).

### Statistical analysis

The collected data for sow performance was analyzed using a t-test with parity and grouping as random effects. To determine the effect of probiotic administration, individual sows and their progenies (until d 21) were used as experimental units. Data represent the standard error of the means and *P* < 0.05 and *P* < 0.10 were set as significant and trends, respectively. The dietary treatment of both experiments was used as fixed effects. The main effect of sow and weaners growth performance (both EXP) and nutrient digestibility, and cytokine immune were analyzed using One-way analysis of variance in a randomized block design, with pen and individual piglets as experiment unit, respectively. Individual feces score was analyzed assuming a binomial distribution, by which scores 1–3 was considered as normal feces and scores 4 and 5 was considered as diarrheas. The data are presented as ± standard error. a, b superscripts in the means denotes significant. Alpha-diversity was determined by observing the ASVs, Chao1 index, Shannon index, and Simpson index, and Pielou_ evenness indices, which account for richness and evenness. Beta-diversity was measured using principal coordinate analysis of both unweighted UniFrac and Bray–Curtis distances. Differential taxonomic markers for each group were determined using the Linear discriminant analysis effect size (LEfSe).

### Supplementary Information

**Additional file 1.** Supplementary Figure.

## Data Availability

The data included in this manuscript are available on reasonable request from the corresponding authors.

## Abbreviations

PWD: Post weaning diarrhea
TNF: Tumor necrosis factor
IL-6: Interleukin-6
LPS: Lipopolysaccharide
BFTL: Backfat thickness loss
BWL: Body weight loss
LEfSe: Linear discriminant analysis effect size
DADA2: Divisive Amplicon Denoising Algorithm 2
ELISA: Enzyme-linked immunosorbent assay
DM: Dry matter
N: Nitrogen
E: Energy
*E. coli*: *Escherichia coli*
