# Correction: Dietary *Bacillus* spp. supplementation to both sow and progenies improved post-weaning growth rate, gut function, and reduce the pro-inflammatory cytokine production in weaners challenged with* Escherichia coli K88*

**DOI:** 10.1186/s42523-024-00302-x

**Published:** 2024-03-19

**Authors:** Vetriselvi Sampath, Sungbo Cho, Jinuk Jeong, Seyoung Mun, Choon Han Lee, Rafael Gustavo Hermes, Apichaya Taechavasonyoo, Natasja Smeets, Susanne Kirwan, Kyudong Han, In Ho Kim

**Affiliations:** 1 Department of Animal Resource and Science, Dankook University, No. 29 Anseodong, Cheonan, Chungnam 330-714 South Korea; 2 Department of Bioconvergence Engineering, Dankook University, Jukjeon, 16890 Republic of Korea; 3 Department of Microbiology, College of Science and Technology, Dankook University, Cheonan, 31116 Republic of Korea; 4 Center for Bio-Medical Engineering Core Facility, Dankook University, Cheonan, 31116 Republic of Korea; 5 Kemin Industries Inc Headquarters, 1900 Scott Ave Des Moines, Des Moines, IA 50317 USA

**Correction: Animal Microbiome (2024) 6:3** 10.1186/s42523-024-00290-y

Following publication of the original article [1], we have been notified that Funding note was published incorrectly.

Now it is:

This research was supported by Basic Science Research Capacity Enhancement Project through Korea Basic Science Institute (National research Facilities and Equipment Center) grant funded by the Ministry of Education (Grant No. 2019R1A6C1010033).

It should be:

This research was supported by Basic Science Research Program through the National Research Foundation of Korea (NRF) funded by the Ministry of Education (NRF-RS-2023-00275307) and Kemin industries (USA).

