# Supplementary material for: Dietary Bacillus spp. supplementation to both sow and progenies improved post-weaning growth rate, gut function, and reduce the pro-inflammatory cytokine production in weaners challenged with Escherichia coli K88
Source: Anim Microbiome. 2024 Jan 24;6:3. doi: 10.1186/s42523-024-00290-y (PMC10809626; doi:10.1186/s42523-024-00290-y)
Supplement: Supplementary file 1 — Additional file 1. Supplementary Figure. [file 42523_2024_290_MOESM1_ESM.pdf]

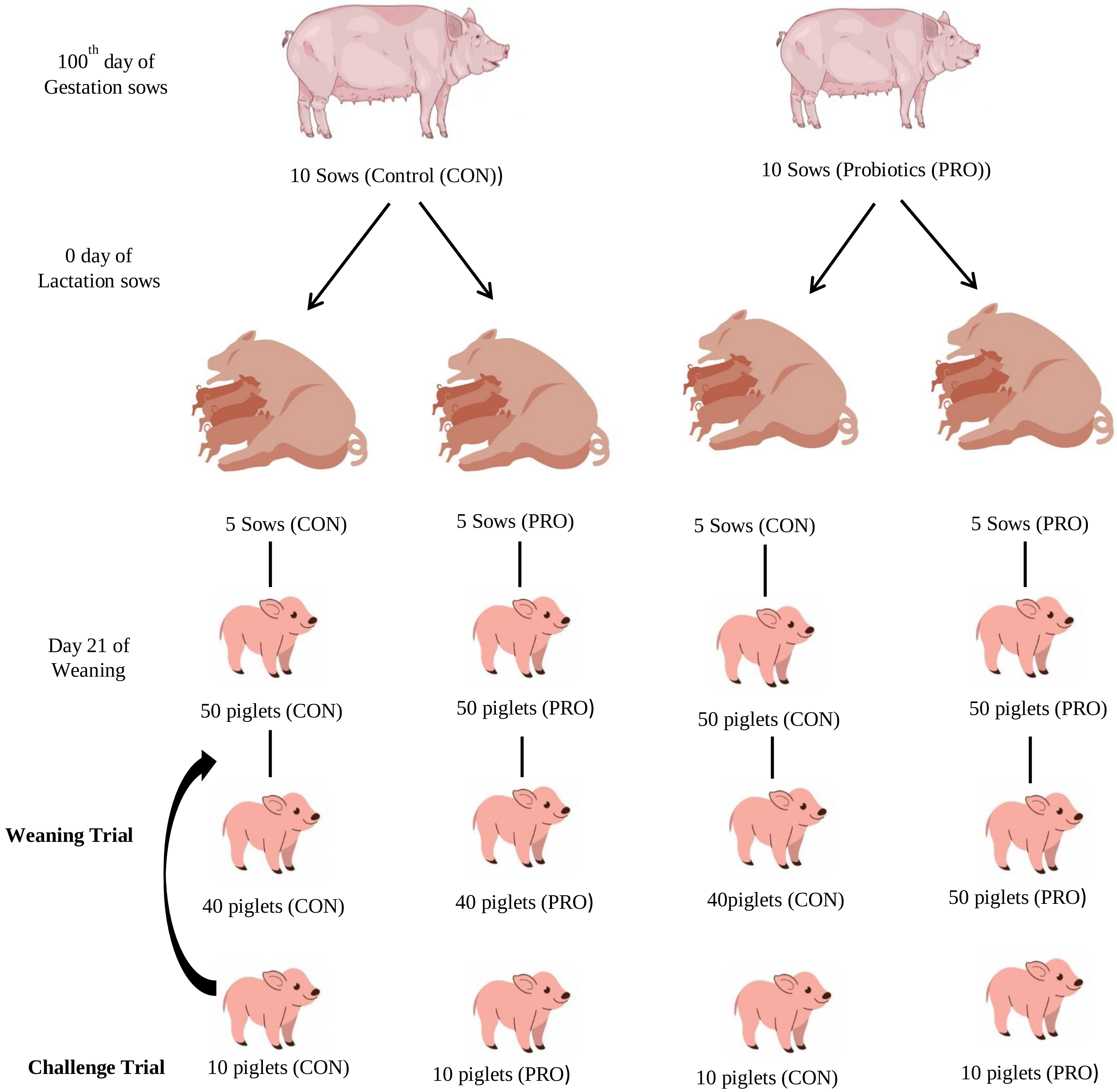
100th day of Gestation sows
10 Sows (Control (CON))
10 Sows (Probiotics (PRO))
0 day of Lactation sows
5 Sows (CON)
5 Sows (PRO)
5 Sows (CON)
5 Sows (PRO)
Day 21 of Weaning
50 piglets (CON)
50 piglets (PRO)
50 piglets (CON)
50 piglets (PRO)
Weaning Trial
40 piglets (CON)
40 piglets (PRO)
40piglets (CON)
50 piglets (PRO)
Challenge Trial
10 piglets (CON)
10 piglets (PRO)
10 piglets (CON)
10 piglets (PRO)
